# Fluorescence Diagnosis in Neurooncology: Retrospective Analysis of 653 Cases

**DOI:** 10.3389/fonc.2019.00830

**Published:** 2019-09-06

**Authors:** Sergey A. Goryaynov, Vladimir A. Okhlopkov, Denis A. Golbin, Konstantin A. Chernyshov, Dmitrij V. Svistov, Boris V. Martynov, Alexandr V. Kim, Vadim A. Byvaltsev, Galina V. Pavlova, Artem Batalov, Nikolay A. Konovalov, Petr V. Zelenkov, Victor B. Loschenov, Alexandr A. Potapov

**Affiliations:** ^1^N. N. Burdenko National Medical Research Center of Neurosurgery of the Ministry of Health of the Russian Federation, Moscow, Russia; ^2^I. M. Sechenov First Moscow State Medical University of the Ministry of Health of the Russian Federation, Moscow, Russia; ^3^S. M. Kirov Military Medical Academy of the Ministry of Defense of the Russian Federation, St-Petersburg, Russia; ^4^V. A. Almazov Federal North-West Medical Research Centre of the Ministry of Health of the Russian Federation, St-Petersburg, Russia; ^5^Laboratory of Neurosurgery, Irkutsk Scientific Center of Surgery and Traumatology, Irkutsk, Russia; ^6^Department of Neurosurgery, Irkutsk State Medical University, Irkutsk, Russia; ^7^Institute of Gene Biology, Russian Academy of Science, Moscow, Russia; ^8^Prokhorov General Physics Institute of the Russian Academy of Science, Moscow, Russia; ^9^National Research Nuclear University MEPhI, Moscow, Russia

**Keywords:** fluorescence diagnosis, 5-Aminolevulinic acid (ALA), glioma, meningioma, neurooncology

## Abstract

**Objective:** This study is to analyze fluorescence sensitivity in the diagnosis of brain and spinal cord tumors.

**Material and methods:** The authors conducted a multicenter retrospective analysis of data on 653 cases in 641 patients: 553 of them had brain tumors and 88 spinal cord tumors. Brain tumor resection was performed in 523 patients, of whom 484 were adults and 39 children. The analyzed series was presented by 320 gliomas, 101 meningiomas, and 72 metastases. A stereotactic biopsy was performed in 20 patients and endoscopic surgery in 10 patients. In all cases, 20 mg/kg of 5–Aminolaevulinic acid was administered orally 2-h before surgery. All surgical interventions were performed with a microscope BLUE 400 to visualize fluorescence, while endoscopic surgery—with an endoscope equipped with a fluorescent module. Fluorescence spectroscopy was conducted in 20 cases of stereotactic biopsies and in 88 cases of spinal cord tumors.

**Results:** Among adult brain tumors operated by microsurgical techniques, meningiomas showed the highest 5-ALA fluorescence sensitivity 94% (*n* = 95/101), brain metastases 84.7% (*n* = 61/72), low-grade gliomas 46.4% (*n* = 26/56), and high-grade gliomas 90.2% (*n* = 238/264). In children the highest 5-ALA visible fluorescence was observed in anaplastic astrocytomas 100% (*n* = 4/4) and in anaplastic ependymomas 100% (*n* = 4/4); in low-grade gliomas it made up 31.8% (*n* = 7/22). As for the spinal cord tumors in adults, the highest sensitivity was demonstrated by glioblastomas 100% (*n* = 4/4) and by meningiomas 100% (*n* = 4/4); Fluorescence was not found in gemangioblastomas (*n* = 0/6) and neurinomas (*n* = 0/4). Fluorescence intensity reached 60% (*n* = 6/10) in endoscopic surgery and 90% (*n* = 18/20) in stereotactic biopsy.

**Conclusion:** 5-ALA fluorescence diagnosis proved to be most sensitive in surgery of HGG and meningioma (90.2 and 94.1%, respectively). Sensitivity in surgery of intracranial metastases and spinal cord tumors was slightly lower (84.7 and 63.6%, correspondingly). The lowest fluorescence sensitivity was marked in pediatric tumors and LGG (50 and 46.4%, correspondingly). Fluorescence diagnosis can also be used in transnasal endoscopic surgery of skull base tumors and in stereotactic biopsy.

## Introduction

In late 90-ies first data on the potential use of 5-ALA in surgery of malignant gliomas have appeared (1, 2). Recently, use of 5-ALA has become more popular for other classes of brain and spinal cord tumors like meningioma (3–7), metastasis (8–11), neurocytoma (12), ependymoma (13, 14), medulloblastoma (15), adenoma (16). Besides microneurosurgery, intraoperative FD has found its application in endoscopic (17–21) and stereotactic surgery (22, 23).

Our study was aimed at analyzing 5-ALA fluorescence sensitivity in patients with brain and spinal cord tumors using microsurgical, endoscopic techniques, along with stereotactic biopsy.

## Materials and Methods

### Patients

The study enrolled 641 patients with brain and spinal cord tumors, operated within 2010–2017 in 3 clinical hospitals: N. N. Burdenko National Medical Research Center of Neurosurgery of the Ministry of Health of the Russian Federation, Moscow; S. M. Kirov Military Medical Academy of the Ministry of Defense of the Russian Federation, St-Petersburg; V. A. Almazov Federal North-West Medical Research Centre of the Ministry of Health of the Russian Federation, St-Petersburg. More data on patients are presented in Table 1. The written informed consent to the surgery and 5-ALA administration was obtained from all patients. The study was approved by the local ethics committee of the N. N. Burdenko National Medical Research Center of Neurosurgery.

**Table 1:** Cases.

**Object**	**5-ALA +**	**5-ALA -**
**Brain tumors, open resection (*n* = 535)**		
Adults (*n* = 493)	420 (85.2%)	73 (14.8%)
Glioma (*n* = 320)	264 (82.5%)	56 (17.5%)
LGG (*n* = 56)	26 (46.4%)	30 (53.6%)
Pilloid astrocytoma	3 (100%)	0 (0%)
Diffuse astrocytoma	6 (25%)	18 (75%)
Gemistocytic astrocytoma	3 (100%)	0 (0%)
Ganglioastrocytoma	0 (0%)	1 (100%)
Non-infantile desmoplastic ganglioglioma	1 (100%)	0 (0%)
Oligoastrocytoma	8 (50%)	8 (50%)
Oligodendroglioma	3 (50%)	3 (50%)
Pleomorphic xanthoastrocytoma	2 (100%)	0 (0%)
HGG (*n* = 264)	238 (90.2%)	26 (9.8%)
Anaplastic astrocytoma	19 (65.5%)	10 (34.5%)
Anaplastic hemangiopericytoma	1 (100%)	0 (0%)
Anaplastic oligoastrocytoma	5 (71.4%)	2 (28.6%)
Anaplastic oligodendroglioma	4 (66.7%)	2 (33.3%)
Glioblastoma (*n* = 211)	203 (94%)	12 (6%)
Gliosarcoma	6 (100%)	0 (0%)
Meningioma (*n* = 101)	95 (94.1%)	6 (5.9%)
Grade I	75 (96.2%)	3 (3.8%)
Grade II	18 (85.7%)	3 (14.3%)
Grade III	2 (100%)	0 (0%)
Brain metastasis (*n* = 72)	61 (84.7%)	11 (15.3%)
Lungs cancer	21 (80.7%)	5 (19.3%)
Breast cancer	18 (90%)	2 (10%)
Other metastases	21 (66.7%)	3 (33.3%)
Pediatric (*n* = 42)	21 (50%)	21 (50%)
LGG (*n* = 22)	7 (31.8%)	15 (68.2%)
Disembrioplastic neuroepithelial tumor	0 (0%)	1 (100%)
Fibrillary protoplasmic astrocytoma	0 (0%)	2 (100%)
Pilocytic astrocytoma	0 (0%)	4 (100%)
Pilomixoid astrpcytoma	4 (57.1%)	3 (42.9%)
Oligoastrocytoma	1 (50%)	1 (50%)
Oligodendroglioma	0 (0%)	1 (100%)
Fibrillary astrocytoma	1 (50%)	1 (50%)
Ependymoma	1 (50%)	1 (50%)
Chorioid papilloma	0 (0%)	1 (100%)
HGG (*n* = 20)	14 (70%)	6 (30%)
Anaplastic astrocytoma	4 (100%)	0 (0%)
Anaplastic ependymoma	4 (100%)	0 (0%)
Glioblastoma	4 (80%)	1 (20%)
Medulloblastoma	1 (16.7%)	5 (83.3%)
Primitive neuroectodermal tumor	1 (100%)	0 (0%)
**Brain tumor, Endoscopic surgery (*****n*** **=** **10)**	6 (60%)	4 (40%)
Meningioma	3 (75%)	1 (25%)
Hordoma	1 (50%)	1 (50%)
Neurinoma	1 (100%)	0 (0%)
Inverted papilloma	1 (100%)	0 (0%)
Other tumors	0 (0%)	2 (100%)
**Brain tumor, stereotactic biopsy (*****n*** **=** **20)**	18 (90%)	2 (10%)
Fibrillary protoplasmic astrocytoma	2 (66.7%)	1 (33.3)
Anaplastic astrocytoma	8 (100%)	0 (0%)
Glioblastoma	7 (100%)	0 (0%)
Other	1 (50%)	1 (50%)
**Adults, spinal cord (*****n*** **=** **88)**	56 (63.6%)	32 (36.4%)
Ependymoma	37 (72.5%)	14 (27.5%)
Astrocytoma	4 (33.3%)	8 (66.7%)
Glioblastoma	4 (100%)	0 (0%)
Gemangioblastoma	0 (0%)	6 (100%)
Meningioma	11 (100%)	0 (0%)
Neurinoma	0 (0%)	4 (100%)
**Overall**	**653**	

### Drugs and Equipment

Having got the informed consent and data on absence of any significant liver or kidney pathology, patients were administered 20 mg/kg of 5–Aminolevulinic acid (“Alasens,” SSC “NIOPIK,” Russian Federation) in 2-h before surgery. Contraindications for “Alasens” were: more than 3-or 4-time ALT, AST increase, porphyria, pregnancy, breastfeeding. In children, 5-ALA was ingested after obtaining the informed parents' consent and by the ethics committee approval. All operations were performed with OPMI Pentero (Carl Zeiss Meditec AG, Obrekochen, Germany) microscope equipped with a fluorescent 400-nm UV light and filters (BLUE 400). During stereotactic surgery MRI (magnetic resonance imaging) and PET (positron emission tomography) with methionine were performed to select the target area and to obtain histological samples.

Operations were performed using the stereotactic system “OREOL” [“Elektropribor,” St-Petersburg; (24)]. All assessments were made on an optic-fiber fluorescence-reflectance multichannel ≪Skin-AGE≫ spectrometer (25). In patients with spinal cord tumors, laser spectroscopy was performed using LESA-01-BIOSPEK spectrum analyzer (laser electronic-spectral device, “Biospek” company, Moscow). The PpIX level between the tumor tissue and the intact brain was measured during spectroscopy.

For endoscopic procedures there was used an endoscope equipped with a fluorescence filter; in a number of cases, the combined video-recording system was applied, which was developed in the Laboratory of Laser biospectroscopy at the A. M. Prokhorov general physics institute of the Russian Academy of Sciences.

## Outcomes of 5-ALA use in Surgery of Brain and Spinal Cord Tumors

### 5-ALA Fluorescence in Surgery of Brain Tumors in Adults (*n* = 493)

Visible fluorescence was marked in 90.2% (*n* = 238/264) of patients with WHO Grade III-IV gliomas and in 46.4% (*n* = 26/56) of patients with WHO Grade I-II gliomas. More data on patient distribution into subgroups by tumor histology are presented in Table 1. Positive fluorescence was marked in 96.2% (*n* = 75/78) of patients with WHO Grade I, in 85.7% (*n* = 18/21) of WHO Grade II, and in 100% (*n* = 2/2) of WHO Grade III meningiomas.

From two to four nodes were revealed in 17 of 63 patients with metastatic brain lesions; overall, 5-ALA fluorescence was measured in 72 tumor cases. Based on tumor location, it was positive in 84.7% (*n* = 61/72) of cases, namely: metastatic lung cancer was positive in 80.7% (*n* = 21/26), metastatic breast cancer in 90% (*n* = 18/20), others in 66.7% (*n* = 21/24).

### 5-ALA Fluorescence in Surgery of Brain and Spinal Cord Tumors in Children (*n* = 42)

In this subgroup 39 of 42 patients were operated (3 patients underwent repeated surgery). Anaplastic astrocytoma and anaplastic ependymoma demonstrated the maximal sensitivity, which made up 100% (*n* = 4/4), each; sensitivity in glioblastomas reached 80% (*n* = 4/5), and in low-grade gliomas, it averaged 31.8% (*n* = 7/22).

It is interesting to note, that medulloblastomas demonstrated “weak” fluorescence (16.7%, *n* = 1/6) despite their high grade of malignancy.

### 5-ALA Fluorescence in Endoscopic Neurosurgery (*n* = 10)

Ten operations have been performed: anterior cranial fossa tumor resection by a transnasal approach. By all that, fluorescence was marked in 75% (*n* = 3/4) of meningiomas, in 50% (*n* = 1/2) of chordomas, in 100% (*n* = 1/1) of neurinoma, and in 100% (*n* = 1/1) of inverted papilloma.

### Fluorescence-Guided Stereotactic Biopsy of Brain Tumors (*n* = 20)

Stereotactic biopsy and stereotactic fluorescence biospectroscopy revealed positive tumor fluorescence in 90% (*n* = 18/20) of cases. Fluorescence spectroscopy was sensitive for detecting fibrillary-protoplasmatic astrocytomas in 66.7% (*n* = 2/3), anaplastic astrocytomas in 100% [(*n* = 8/8), glioblastomas in 100% (*n* = 7/7)] of cases. The false-positive effect was marked in 5.6% (*n* = 1/18) of patients (reactive gliosis after earlier performed radiotherapy).

### 5-ALA Fluorescence in Surgery of Spinal Cord Tumors in Adults (*n* = 88)

Visible fluorescence was marked in 75% (*n* = 33/44) of intramedullary ependymomas, wherein, a strong PpIX fluorescence, revealed by laser spectroscopy, was observed in 100% (*n* = 44/44) of cases. Astrocytomas have been noted to fluoresce in 33.3% (*n* = 4/12) of cases, spectroscopy has demonstrated PpIX fluorescence in 50% (*n* = 6/12). The highest fluorescence sensitivity was marked in patients with glioblastoma (4/4) and meningioma (11/11). Fluorescence was visible in 57.1% (*n* = 4/7) of cauda equine ependymoma; PpIX fluorescence intensity, revealed by laser spectroscopy, was (*n* = 7/7) in all cases; negative PpIX fluorescence was observed in hemangioblastoma (*n* = 0/6) and neurinoma (*n* = 0/4).

## Discussion

At the end of 90-ies first data on the potential use of 5-ALA in neurosurgery appeared, with first publications being devoted to malignant gliomas (1, 22). Today, 5-ALA is applied in surgery of different types of brain and spinal cord tumors for both adults and children, including meningiomas (3–7, 26–28), metastasis (8–11), neurocytomas (12), ependymomas (13, 14), medulloblastomas (15).

Among the publications devoted to application of 5-ALA fluorescence in surgery of CNS tumors, Marbacher et al. (10) (*n* = 458) reports the largest series of patients (10). In contrast to Marbacher et al. (10), our series (*n* = 641) enrolled patients with spinal cord tumors (*n* = 88), children (*n* = 39), and patients who underwent transnasal endoscopic surgery (*n* = 10). The novelty of our research is application of spectroscopy in cases of spinal cord tumors (*n* = 88) and stereotactic biopsy (*n* = 20).

We found it reasonable to combine data on intraoperative fluorescence sensitivity obtained in different tumors of the CNS. In our series, to visualize fluorescence in stereotactic biopsy and in spinal surgery, traditionally, besides microscope, the spectroscopic control is used.

### Adults, Brain, Open Resection

#### High-Grade Gliomas

A number of papers have proved that use of fluorescence diagnosis in surgery of high-grade gliomas results in the increased rate of GTR (gross total resection) and the increased progression-free and overall survival time (29–32). Thus, use of fluorescence diagnosis in surgery of high-grade gliomas is an effective technique.

The results of 5-ALA application in adults with different types of brain tumors were highlighted in detail in our earlier publications (18, 18, 21, 26). In this study, fluorescence sensitivity in the treatment of high-grade gliomas was 90.2% (*n* = 238/264), which corresponded to findings of other authors (22, 32–34).

#### Low-Grade Gliomas

Whether it is reasonable to use 5-ALA in surgery of non-contrasting gliomas, is still undefined. On the one hand, it is evident knowledge that gross total resection results in the increased progression-free and overall survival time among patients with LGG (31). However, no research has proved that use of fluorescence in LGG results in the increased rate of GTR. For these reasons, there is a strong need for further studying and specifying this problem.

Widhalm et al. have reported that PpIX accumulates in anaplastic focus, where the tumor has higher grade areas of malignancy (Grade III) (23). According to our data, fluorescing focuses in LGG are areas with the Ki 67 index still not exceeding 5% (35). Medical publications provide contradictory opinions on fluorescence sensitivity in LGG, which varies from 7.7 to 40% (10, 36–38). In our group it made up 46.4% (*n* = 26/56), presumably due to comprising different types of gliomas; while the lowest sensitivity was observed in diffuse astrocytomas 25% (*n* = 6/18).

#### Metastases

**Figure 1:**
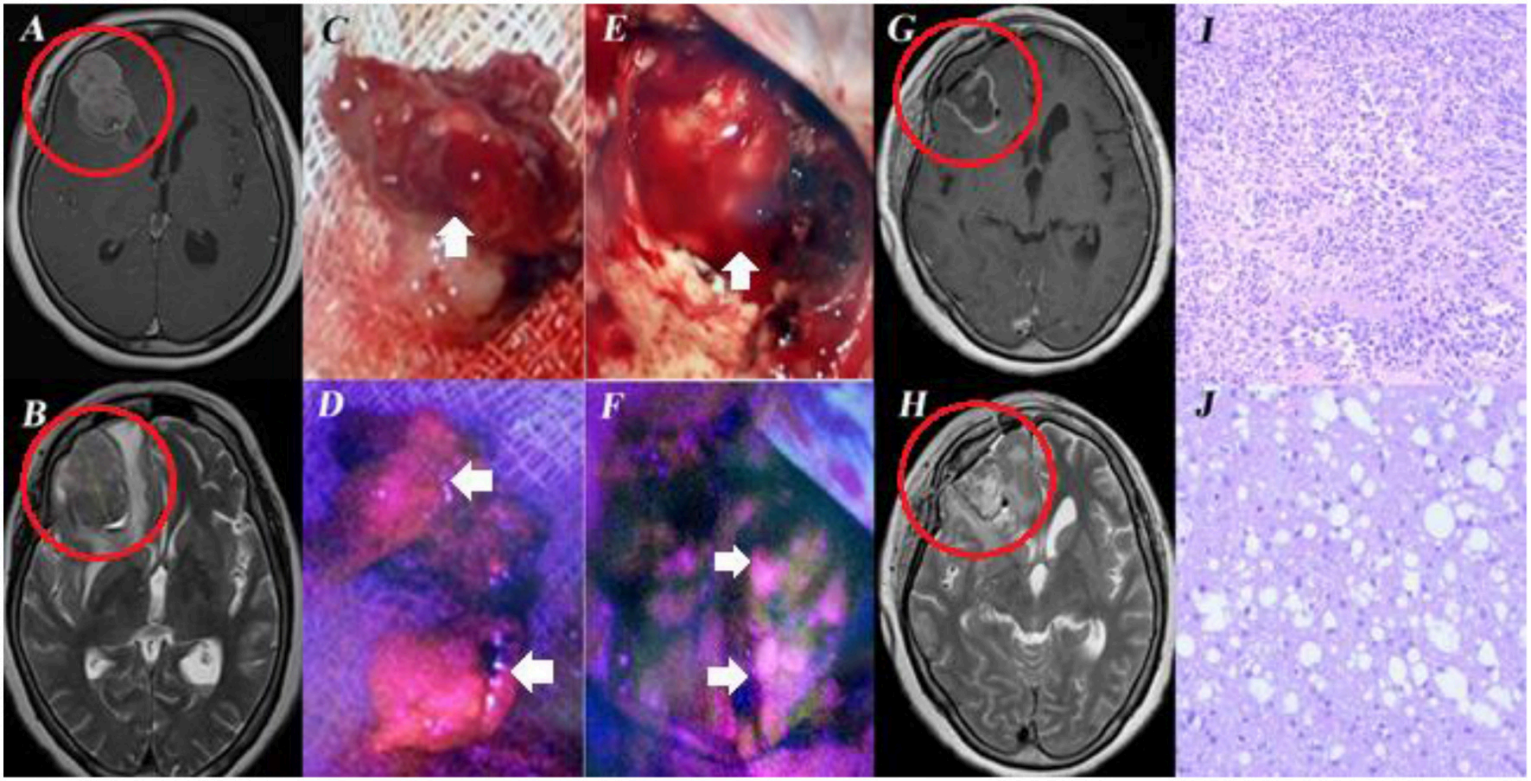
Female, aged 60, admitted to the clinical center with complaints of headaches. In 2009 melanoma of the right lumbar region was removed, in 2017 she underwent resection of the superior lobe of the left lung. MRI of the brain revealed an intracerebral tumor in the right frontal lobe with perifocal edema **(A,B)**. Resection of the right frontal lobe tumor was performed under 5-ALA Fluorescence assistance. During surgery, bright fluorescence of the tumor node was marked **(C,D)**. Biopsy revealed melanoma metastasis **(I)**. immunohistochemical examination showed positive HMB45, MelA expression. After resection, the unchanged white matter of the frontal lobe was exposed in the tumor bed **(E)**. Visible tumor bed fluorescence was observed **(F)**. No tumor tissue was revealed by additional biopsy samples taken from the fluorescing region **(J)**. Postoperative MRI **(G,H)** demonstrated total tumor node resection. This case is interesting, first, by the fact that melanoma metastasis can fluorescence and second, that the resected tumor bed without tumor cells can also fluoresce. Informed consent has been obtained from the patient for the publication of data, including images.

In our series, 5-ALA-induced fluorescence for cerebral metastases was 84.7% (*n* = 61/72), which is slightly higher compared to other studies. Thus, according to Kamp and co-authors, fluorescence sensitivity for cerebral metastases varied from 52 to 81% (11). The absence of fluorescence in intracranial metastases indicated a significantly higher incidence of local tumor progression after surgery (11). It might be related to different surgeon's experience in using 5-ALA, which is particularly important when dealing with weak fluorescence intensity. The phenomenon of tumor bed, still fluorescing after metastatic node removal, is still hard to understand (clinical case, Figure 1).

#### Meningiomas

These data prove our previous research on 5-ALA sensitivity in intracranial meningioma surgery (39).

### Children, Brain, Open Resection

As was earlier mentioned, use of fluorescence diagnosis in HGG in children results in the increased rate of GTR (gross total resection) and the increased progression-free and overall survival time (40, 41). New data on the clinical use of 5-ALA in children with some other types of brain tumors have been reported (15, 24, 41–43). Thus, fluorescence diagnosis is recommended for HGG. For other classes of tumors, its use is disputable (44).

In our series, fluorescence sensitivity in children with brain tumors was 50% (*n* = 21/42) (45), thus corresponding to other foreign authors' experience (24, 41, 43, 46). Fluorescence sensitivity depends on the tumor morphology and malignancy. According to Stummer et al. fluorescence sensitivity was noted to make up 15% (*n* = 2/13) for Grade I-II astrocytoma, and 85% (*n* = 12/14)—for glioblastoma. In our research, florescence sensitivity for low-grade gliomas was 31.8% (*n* = 7/22), for high-grade gliomas −70% (*n* = 14/20). Maximal sensitivity was revealed in anaplastic astrocytomas 100% (*n* = 4/4) and anaplastic ependymoma 100% (*n* = 4/4).

Still confusing is, that medulloblastomas in children show low fluorescence sensitivity. This high-grade class of tumors, however, reveals “weak” fluorescence (30). For instance, Beez et al. report fluorescence sensitivity reaching 25% (*n* = 1/3) (43). In our series, it made up 16.7% (*n* = 1/6) for medulloblastomas. As Zhang et al. reports, more complete resection rate with the following increased survival time was associated with positive fluorescence (44).

### Adults, Spinal Cord

5-ALA fluorescence-assisted technology in adults has proved to be a good method of intraoperative neuroimaging (13, 14, 47–49). In our experience, fluorescence sensitivity for spinal cord tumors was 63.6% (*n* = 56/88), which was similar to other publications (13, 14, 48). Millesi et al. report fluorescence sensitivity to range from 0% (*n* = 0/8) in neurinomas to 100% (*n* = 12/12) in meningiomas (48), thus, also confiding with our data. Application of spectroscopy in our series allowed us to increase fluorescence sensitivity in astrocytomas (Grade II) from 33.3% (*n* = 4/12) to 50% (*n* = 6/12), and in ependymomas from 75% (*n* = 33/44) to 100% (*n* = 44/44), including cauda equine ependymoma, the sensitivity of which varied from 57.1% (*n* = 4/7) to 100% (7/7).

### Endoscopic Surgery

The present-day microscope-assisted metabolic navigation possesses the insufficient quality of the fluorescence signal obtained during a deep and narrow approach. To solve this problem, a specific design of fluorescence endoscope has been adopted. 5-ALA fluorescence is known to be useful in visualizing the tumor tissue (18, 19, 50, 51), while fluorescence with indocyanine green (ICG)—for coloration of the vessels (52–55).

There are only a few medical publications devoted to endoscopic surgery with fluorescence assistance in neurooncology. Takeda et al. reported 2 cases of germinomas (56), in which 5-ALA fluorescence-guided endoscopic surgery was used. Cornelius et al. described the procedure of using 5-ALA endoscopic surgery in 2 patients with meningiomas (57). Our series included 10 patients with different skull base tumors; intraoperative fluorescence was visualized in 60% of cases. Alongside with this, meningiomas was visualized in 3 cases out of 4, chordoma in 1 case out of 2, neurinoma in 1 case of 1, inverted papilloma in 1 case of 1.

Patients who underwent microscopic 5-ALA fluorescence-assisted endoscopic surgery for brain tumors did not enter this series. These results were reported in our early publication in 2008 (18). We did not use 5-ALA fluorescence in transventricular endoscopic surgery of intraventricular tumors. Takeda et al. have earlier reported on that (56).

### Stereotactic Biopsy

According to the world literature databank, STB proved to be not informative in 24% (58). There are three main methods neurosurgeons have for reduction the incidence of uninformative biopsies: a combination of MRI and PET is used for preoperative planning, biopsy in the operating room and the use 5-ALA fluorescence. A fluorescence-assisted stereotactic biopsy can be obtained in 2 ways: first, by measuring the concentration of PP IX in the tumor tissue along the stereotactic trajectory to the target using a spectral probe before extracting the biopsy (59). Second, by evaluating the tissue sample fluorescence using a neurosurgical microscope with a special fluorescence regimen, as described by Widhalm et al. (23). In the latter, specimens were put under fluorescence light of a surgical microscope to assess their fluorescence. Fluorescence sensitivity, according to Widhalm's report, was 100% for HGG and 0% for LGG. In addition, use of fluorescence was helpful in shortening the operation time and decreasing the average number of biopsies during surgery (60).

In our opinion, the combination of the stereotactic cannula and the spectral probe is easier and does not require an operative microscope with a fluorescence module. Stereotactic biopsy combined with fluorescence spectroscopy gives a chance when analyzing, to determine the zones of the highest concentration of PPIX and select the tumor site with the highest degree of anaplasia, thus increasing the diagnostic value of stereotactic biopsy as a technique (23, 61).

Fluorescence sensitivity in our series reached 90% (*n* = 18/20), thus corresponding to the world literature data. Marbacher et al. (10) reports fluorescence sensitivity of 88% (*n* = 44/50). Fluorescence sensitivity for a low-grade glioma, particularly for fibrillary protoplasmic glioma, was 66.7% (2/3), while in the study of Marbacher et al. it was 25% (3/12). In our experience, higher fluorescence can be explained by using stereotactic biopsy under spectroscopic, and not only visual, control. A small number of cases in our series does not allow us to perform the comparative analysis of fluorescence sensitivity for patients with low-grade glioma in the STB subgroup.

## Conclusion

5-ALA fluorescence diagnosis proved to be most sensitive in surgery of HGG and meningioma (90.2 and 94.1%, respectively). Sensitivity in surgery of intracranial metastases and spinal cord tumors was slightly lower (84.7 and 63.6%, correspondingly). The lowest fluorescence sensitivity was marked in pediatric tumors and LGG (50 and 46.4%, correspondingly). Fluorescence diagnosis can also be used in transnasal endoscopic surgery of skull base tumors and in stereotactic biopsy.

## Limitations

There are several limitations our work has. Firstly, we used brain tumor classification of the 2007 year (because it is a retrospective study). Secondly, it common knowledge that medulloblastomas (MB) are recommended to classify into subtypes, according to WHO classification, but our analysis was performed earlier than an official MB sub-type classification was generally accepted. In addition, MB sub-type classification is effective for a traditional classification of patients into moderate or high-grade risk groups and is important for further prognosis and choice of the differentiated treatment modality. In our opinion, the practical value of such a comparative analysis is not so important, because fluorescence degree provides an increase in the radical blastomatous tissue resection rate and not assessment of its molecular genetic features. Our group of MB patients is too small to claim any novelty. We just state that the data obtained can be helpful for a meta-analysis.

## Ethics Statement

All procedures performed in studies involving human participants were in accordance with the ethical standards of the institutional and/or national research committee and with the 1964 Helsinki declaration and its later amendments or comparable ethical standards.

## Author's Note

AK is a corresponding member of RAS. AP is a full member of RAS.

## Author Contributions

SG and AP: research concept and design. VO, DG, DS, BM, AK, NK, PZ, AP, and AB: data collection and processing. KC: statistic support and writing text. VB, GP, and VL: editing. This work was supported by the ethical committee of Burdenko Neurosurgical Institute.

### Conflict of Interest Statement

The authors declare that the research was conducted in the absence of any commercial or financial relationships that could be construed as a potential conflict of interest.

## Abbreviations

5-ALA: 5–Aminolevulinic acid
FD: Fluorescence diagnosis
HGG: High-grade gliomas (WHO Grades III-IV)
LGG: Low-grade gliomas (WHO Grades I-II)
PpIX: Protoporphyrin IX
PET: Positron Emission Tomography
PDD: Photodynamic diagnosis
STB: Stereotactic Biopsy.
